# COVID-19 vaccination in pregnancy: efficacy of second-generation vaccines, maternal-neonatal safety, and strategies to address vaccine hesitancy

**DOI:** 10.3389/fmed.2026.1835847

**Published:** 2026-06-03

**Authors:** Jia Xu, Fang Liu, Han-Ying Yuan, Heng Pan, Xuan Xi, Che Chen, De-Hong Li, Yan Lu

**Affiliations:** 1 School of Public Health, Gansu University of Chinese Medicine, Lanzhou, Gansu, China; 2 Department of Blood Transfusion, Gansu Provincial Hospital, Lanzhou, China; 3 Department of Clinical Laboratory, Gansu Provincial Hospital, Lanzhou, China

**Keywords:** maternal and neonatal health, Omicron variants, pregnancy, second-generation COVID-19 vaccines, vaccine hesitancy

## Abstract

The COVID-19 pandemic, caused by severe acute respiratory syndrome coronavirus 2 (SARS-CoV-2), has had a profound impact on global public health. The Omicron variant, marked by high transmissibility and pronounced immune evasion capabilities, poses a particularly severe threat. Pregnant women undergo physiological immune modulation during pregnancy, which increases their susceptibility to severe disease following SARS-CoV-2 infection. This heightened risk is evidenced by elevated rates of intensive care unit (ICU) admission and increased maternal mortality. Additional pregnancy-related physiological changes—including heightened oxygen consumption and reduced respiratory reserve—further compromise respiratory function and worsen clinical outcomes in infected individuals. Although robust evidence confirms the efficacy and safety of COVID-19 vaccines, vaccine hesitancy remains prevalent among pregnant women due to concerns about potential adverse effects and fetal safety. This review evaluates current vaccination strategies for pregnant women using second-generation COVID-19 vaccines, with particular emphasis on maternal and neonatal health outcomes. It also analyzes barriers to vaccine uptake and underscores the need for further research to refine vaccination protocols, support equitable access, and ensure the safe and effective deployment of vaccines—ultimately mitigating the risks of SARS-CoV-2 infection during pregnancy.

## 1 Introduction

COVID-19, caused by severe acute respiratory syndrome coronavirus 2 (SARS-CoV-2), emerged in late 2019 and soon became a major global public health challenge (1). Since then, SARS-CoV-2 has diversified continuously. Omicron and its descendant lineages dominated much of the later pandemic, and more recent circulation has been shaped by Omicron-derived sublineages with enhanced transmissibility and substantial immune-evasive capacity (2, 3). Accumulating mutations in the receptor-binding domain and N-terminal domain of the spike protein have weakened neutralization induced by prior infection or earlier vaccines, thereby facilitating immune escape and sustained transmission, including through asymptomatic or mildly symptomatic infections (2, 4). Although Omicron-lineage infection shows greater upper-airway than lower-airway tropism compared with earlier epidemic strains, this does not eliminate the risk of severe disease in populations that remain clinically vulnerable (5).

Among these populations, pregnant women warrant particular attention because of the physiological and immunological adaptations of pregnancy (5). Pregnancy-related immune modulation, together with increased cardiopulmonary demands, may increase susceptibility to severe COVID-19 and is associated with a higher risk of adverse obstetric outcomes, including preeclampsia, preterm birth, and emergency cesarean delivery (6, 7). At the same time, newborns and young infants rely largely on maternally derived protection during early life, when active immune defenses remain immature; therefore, prevention during pregnancy is relevant not only to maternal health but also to protection in early infancy (8). Against a background of ongoing viral evolution and changing population immunity, more targeted vaccination strategies for pregnant women have become increasingly important.

**Figure 1:**
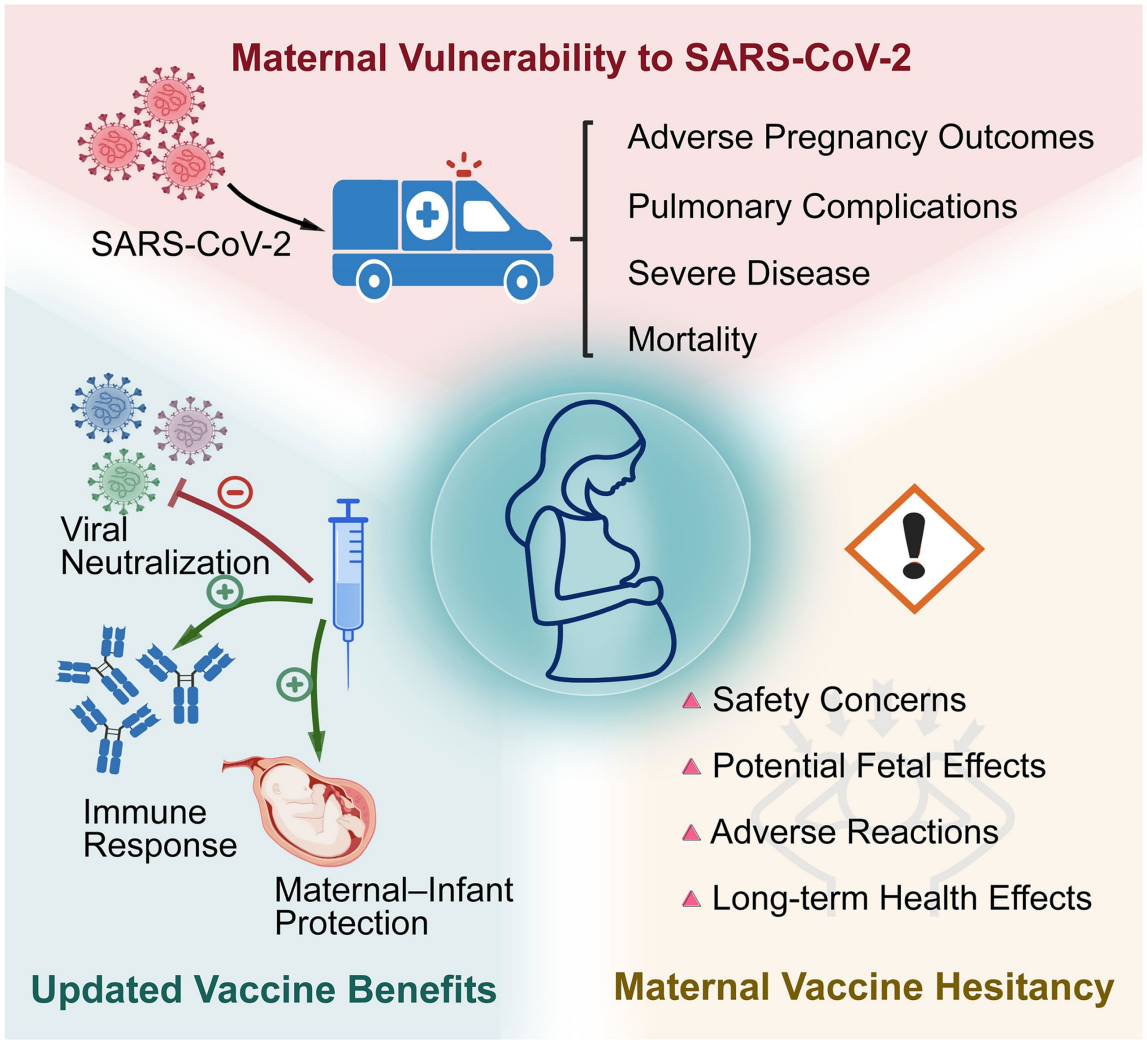
Second-generation COVID-19 vaccines in pregnancy. Overview of maternal vulnerability to SARS-CoV-2, the benefits of second-generation COVID-19 vaccines during pregnancy, and the main factors associated with vaccine hesitancy. Created with BioGDP.com (29).

In this context, second-generation and updated COVID-19 vaccines targeting Omicron-related lineages are of particular interest. For clarity, the term “second-generation vaccines” is used in this review in a broad sense to encompass Omicron-containing bivalent vaccines and later updated formulations, including those based on lineages such as XBB.1.5 and JN.1. Although current evidence broadly supports the effectiveness and safety of COVID-19 vaccination during pregnancy, concerns about fetal safety, pregnancy outcomes, and the adequacy of pregnancy-specific evidence still contribute to vaccine hesitancy. This review summarizes current evidence on the effectiveness, immunogenicity, and maternal and neonatal safety of second-generation COVID-19 vaccines in pregnancy and discusses potential approaches to improving vaccine acceptance in this population (Figure 1).

## 2 Pregnancy vulnerability and vaccination rationale

Pregnancy is characterized by physiological and immunological changes that may alter maternal responses to SARS-CoV-2 infection (9). The increased cardiopulmonary burden of gestation may further worsen adverse clinical outcomes. Maternal immune responses during pregnancy are influenced by genetic background, maternal age, pre-existing comorbidities, and gestational adaptations (9, 10). To maintain maternal-fetal tolerance, pregnancy involves complex immune regulation together with substantial cardiopulmonary changes, including increased circulatory and respiratory demand, diaphragmatic elevation, greater oxygen consumption, and airway mucosal edema (11, 12). These changes should not be viewed simply as immunosuppression; rather, they reshape host responses to viral infection and may increase the risk of severe COVID-19, serious pulmonary complications, and adverse pregnancy outcomes (9, 11). One study found that 27.5, 27.0, and 45.5% of Omicron infections in pregnant women occurred during the first, second, and third trimesters, respectively (11). Compared with nonpregnant women of reproductive age, pregnant women with COVID-19 were reported to be three times more likely to require intensive care unit (ICU) admission, 2.4 times more likely to receive extracorporeal membrane oxygenation (ECMO), and 1.7 times more likely to die (10). These observations support prioritizing pregnant women in COVID-19 prevention and vaccination strategies.

In this setting, vaccination during pregnancy offers benefits beyond reducing the risk of severe maternal disease; it also provides early passive protection to the newborn through transplacental antibody transfer (8). Available evidence indicates that COVID-19 vaccination in pregnancy elicits sustained neutralizing antibody responses in maternal serum, with functionally relevant antibodies also detectable in cord blood, supporting protection for both mother and infant (13). However, first-generation mRNA vaccines based on the ancestral strain, including mRNA-1273 and BNT162b2, show reduced neutralizing activity against Omicron and its subvariants, indicating that vaccination strategies in pregnancy should be updated as circulating strains continue to evolve (13, 14). Whether second-generation COVID-19 vaccines can provide broader maternal protection and induce more durable immune responses during pregnancy therefore remains an important question.

## 3 Updated COVID-19 vaccines in pregnancy: current evidence

### 3.1 Clinical protection and vaccination timing

As updated COVID-19 vaccines have become available, increasing attention has focused on their clinical effectiveness and timing during pregnancy. In September 2023, the Advisory Committee on Immunization Practices (ACIP) recommended a single dose of the 2023–2024 updated monovalent XBB.1.5 COVID-19 vaccine for all persons aged 6 months and older (15). Observational evidence indicates that vaccination during pregnancy reduces the risk of severe maternal COVID-19 and is associated with lower rates of selected adverse birth outcomes (7, 16). During the Omicron period, first-generation vaccines still provided protection against severe illness, referral to higher-level care, intensive care unit admission, and death, although effectiveness waned over time and with changes in circulating strains (17). Data from the same period also suggested a higher risk of neonatal death among infants born to unvaccinated mothers, whereas maternal vaccination was associated with lower rates of preterm birth and adverse neonatal outcomes (17).

Compared with first-generation vaccines, bivalent and other updated vaccines targeting Omicron-related lineages are more closely aligned with current prevention needs (13). In pregnant persons, administration of a bivalent mRNA vaccine during pregnancy provided greater protection against COVID-19–associated emergency department or urgent care visits than vaccination completed at least 6 months before pregnancy, while vaccination within 6 months before pregnancy retained partial protection (18). Epidemiologic studies further suggest that maternal mRNA vaccination during pregnancy reduces the risk of COVID-19-related hospitalization during the first 6 months of life, although this protection declines over time (8). Available surveillance data indicate that the safety profile of bivalent mRNA vaccines in pregnancy is comparable to that of monovalent mRNA booster doses, with no new or unexpected safety signals identified (16). In addition, available data have not identified new safety concerns with coadministration of COVID-19 and influenza vaccines, whereas pregnancy-specific evidence on coadministration with Tdap remains limited (16, 18). Overall, second-generation vaccines continue to offer meaningful protection during Omicron-related circulation.

### 3.2 Immune response and variant coverage

Understanding how second-generation COVID-19 vaccines shape immune responses and coverage against circulating variants during pregnancy is important for interpreting their clinical relevance. Neutralizing antibody titers are widely regarded as a useful correlate of protection against SARS-CoV-2 infection (19). Available evidence suggests that, compared with pregnant women previously immunized with monovalent AZD1222-containing regimens, those who received a bivalent or updated COVID-19 vaccine showed greater maternal and cord blood neutralizing activity against Omicron-related variants, including BA.5, BF.7, BQ.1, and XBB.1.5 (6, 20). Maternal age and body mass index were not significantly associated with neutralizing inhibition rates in maternal or cord blood, although this finding should be interpreted cautiously given the limited sample size (6). Another study reported higher maternal and cord blood antibody titers when XBB.1.5 vaccination was administered at 28–34 weeks of gestation, suggesting that gestational timing may influence antibody magnitude and transplacental transfer (20).

However, current evidence also indicates that the breadth of vaccine-induced immunity during pregnancy may remain restricted (21). One recent study found that booster vaccination induced IgG responses to circulating variants in pregnant women, but cross-reactivity and neutralizing capacity against newly emerged sublineages such as XBB.1.5 and JN.1 were lower than those observed in nonpregnant women (21). These findings support the immunogenicity of bivalent and updated vaccines during pregnancy, while also suggesting that protection against newly emerging sublineages may be less broad than desired. Because direct head-to-head studies comparing bivalent with first-generation COVID-19 vaccines in pregnant women remain limited, their relative advantages still require confirmation in prospective studies.

### 3.3 Placental transfer and neonatal benefit

Transplacental transfer of vaccine-induced antibodies is a key mechanism of neonatal protection. Because infants younger than 6 months are below the current minimum age for COVID-19 vaccination, early-life protection depends largely on maternally derived antibodies (15). COVID-19 vaccination during pregnancy induces a maternal antibody response and confers passive immunity to the newborn via transplacental antibody transfer (8, 20). Epidemiologic evidence indicates that infants born to mothers who completed a two-dose mRNA vaccine series during pregnancy had an approximately 61% lower risk of COVID-19–related hospitalization in the first 6 months of life (8).

This maternal–infant antibody transfer pathway appears to be preserved after second-generation COVID-19 vaccination (20). Studies have shown that bivalent and XBB.1.5 updated vaccines administered during pregnancy elicit neutralizing activity against Omicron-related sublineages in both maternal and cord blood (6, 20). Neutralizing antibody inhibition rates against several Omicron subvariants exceeded 89% in maternal blood and 82% in cord blood, and remained high against XBB.1.5, reaching 90.57 and 83.69%, respectively (6). An XBB.1.5 vaccine study further reported higher SARS-CoV-2 neutralizing antibody levels in cord blood than in maternal blood, with levels of 3,120 AU/mL and 1,860 AU/mL, respectively, corresponding to an approximately 1.68-fold difference (20). Together, these findings indicate that neutralizing antibodies induced by second-generation vaccines can be transferred across the placenta and may provide newborns with immune protection more relevant to currently circulating variants. Antibody transfer efficiency may also depend on vaccination timing. A longer interval between vaccination and delivery may favor antibody accumulation and transplacental transfer, whereas delivery shortly after vaccination may limit antibody levels in the newborn (6). Some studies have proposed vaccination closer to delivery to maximize passive infant protection, but this should be interpreted cautiously as a study-based strategy rather than a universal recommendation, because current guidance allows COVID-19 vaccination at any stage of pregnancy (18). Overall, second-generation COVID-19 vaccination during pregnancy may support early passive protection in newborns through placental antibody transfer, although the duration of this protection and its effectiveness against newly emerging variants remain uncertain.

### 3.4 Reactogenicity profile and pregnancy safety

Adverse events following second-generation COVID-19 vaccination in pregnant women can be broadly divided into two categories: common post-vaccination reactions and pregnancy-specific outcomes (16, 20). The pattern of common adverse reactions is generally similar to that observed after first-generation vaccines and mainly includes local and systemic symptoms (22). Local reactions most commonly include injection-site pain, erythema, swelling, and occasionally rash, whereas systemic reactions include fever, myalgia, arthralgia, headache, nausea, and vomiting (20). These reactions are usually transient and self-limited and can generally be managed with routine clinical care (23). However, safety assessment in pregnancy should focus not only on reactogenicity but also on clinically relevant obstetric and fetal outcomes, such as spontaneous abortion, stillbirth, placental abnormalities, preterm birth, and fetal growth restriction, which are central to patient counseling and vaccination decision-making (16).

Currently, direct safety evidence for second-generation COVID-19 vaccines in pregnant women comes mainly from post-authorization passive surveillance. In a VAERS-based study, 136 adverse event reports were identified after bivalent mRNA COVID-19 vaccination during pregnancy. The most frequently reported pregnancy-specific event was spontaneous abortion (12 reports, 8.8%), followed by placental abnormalities (8 reports, 5.9%) and preterm birth (6 reports, 4.4%) (16). Spontaneous abortion accounted for approximately one-tenth of pregnancy-related reports, a smaller proportion than that observed after monovalent mRNA booster vaccination in the same analysis and the approximately one-quarter proportion reported to VAERS after the primary COVID-19 vaccine series (16, 24). Under comparable surveillance criteria, pregnancy-specific adverse events were not reported more frequently after bivalent vaccination than after monovalent mRNA booster vaccination (41 reports, 30.1% vs. 154 reports, 36.1%) (16). However, VAERS data reflect reporting patterns rather than true incidence and cannot establish causality. Outcomes such as spontaneous abortion should therefore be interpreted in relation to maternal age, previous pregnancy history, underlying conditions, and background population rates (25). Accordingly, current second-generation vaccine-specific data remain insufficient to directly estimate ORs or RRs for spontaneous abortion, stillbirth, preterm birth, fetal growth restriction, congenital anomalies, or neonatal outcomes. Overall, available bivalent vaccine-specific surveillance data have not suggested new or more frequent pregnancy safety signals compared with monovalent mRNA boosters. For later updated vaccines, prospective, large-scale, formulation-specific studies are still needed to provide outcome-specific risk estimates and improve the precision of counseling during pregnancy.

## 4 Hesitancy drivers and acceptance strategies

Although current evidence and professional guidance support the safety of COVID-19 vaccination for pregnant women and the fetus, hesitancy remains common in this population (26). This hesitancy is shaped by pregnancy-specific risk perceptions, including concerns about spontaneous abortion, fetal harm, long-term child development, and fertility. Some women therefore choose to postpone vaccination until after delivery (7, 27). The underrepresentation of pregnant women in early vaccine trials may also have reduced confidence in subsequent observational studies and post-marketing safety data among some patients (22, 28). Strategies to improve vaccine acceptance should move beyond broad reassurance and provide clear, verifiable, gestational-age-specific information on the risks and benefits most relevant to pregnancy.

Survey findings suggest that physicians are the most trusted source of vaccine information among pregnant women, followed by the Centers for Disease Control and Prevention (CDC), highlighting the importance of professional recommendations in maternal vaccine decision-making (7). Nevertheless, hesitancy during pregnancy is not merely a communication issue. It is shaped by safety concerns, family opinions, misinformation, trust in healthcare systems, and practical barriers to vaccination. Interventions should therefore extend beyond routine clinic-based counseling. Clinical conversations should present outcome-specific evidence on spontaneous abortion, stillbirth, preterm birth, congenital anomalies, neonatal hospitalization, and passive infant protection, while considering gestational age, underlying medical conditions, prior SARS-CoV-2 infection, and vaccination history (16). When appropriate, partners and key family members may be included in antenatal visits or prenatal education classes to address family-level misconceptions (26). Healthcare systems can further support vaccine uptake by ensuring consistent messages from obstetricians, midwives, nurses, and pharmacists and by reducing logistical barriers through simplified scheduling, fewer referral steps, and same-visit vaccination during antenatal care (26). At the community and policy levels, multilingual and culturally tailored materials, community health workers, prenatal education platforms, and peer experience sharing may help reduce misinformation and medical mistrust. Transparent reporting of pregnancy-specific safety and effectiveness data for bivalent and later updated vaccines is also essential (7). Overall, improving COVID-19 vaccine acceptance during pregnancy requires a multilevel approach that combines individualized counseling, family engagement, integration into antenatal care, community trust-building, and data transparency.

## 5 Discussion

Against the continued circulation of Omicron-related lineages, COVID-19 prevention in pregnancy should no longer depend on vaccination strategies based solely on the ancestral strain. Current evidence suggests that second-generation and later updated vaccines are better matched to the contemporary viral landscape, helping to reduce severe disease in pregnant women while also providing early passive protection to infants through transplacental antibody transfer. To date, available data have not identified clear pregnancy safety signals after updated vaccination, and the overall safety profile appears comparable to that of earlier mRNA booster doses, supporting the continued clinical relevance of these vaccines in pregnancy.

However, the evidence base for second-generation COVID-19 vaccines in pregnancy remains limited. Most available inferences are drawn from observational studies and post-marketing surveillance data, and direct comparative evidence across formulations remains sparse, particularly high-quality head-to-head studies comparing earlier bivalent products with later strain-updated vaccines. Immunogenicity data provide important mechanistic support, but they do not by themselves establish the magnitude or durability of clinical protection for mothers or infants. In addition, current vaccine-specific safety data remain insufficient to directly estimate outcome-specific ORs or RRs for key maternal and neonatal outcomes. Continued viral evolution may further narrow the breadth of vaccine-induced immunity and reduce the duration of passive protection in infants, making ongoing evaluation of real-world effectiveness against newly emerging sublineages essential. More prospective comparative studies focused on clinically meaningful maternal and neonatal outcomes are therefore needed to strengthen the evidence base. At the same time, emerging evidence must be translated into guidance that is clear, credible, and clinically actionable. This remains especially important because decisions about vaccination during pregnancy continue to be shaped by concerns about its necessity, fetal safety, and possible longer-term effects, while vaccine uptake in many settings remains suboptimal. Clear communication during antenatal care, reinforced outpatient counseling, and consistent recommendations from healthcare professionals remain essential for informed vaccine uptake during pregnancy. However, acceptance strategies should go beyond clinician-led counseling by integrating outcome-specific risk communication, family engagement, antenatal-care delivery, community trust-building, and transparent reporting of pregnancy-specific safety and effectiveness data. Together, stronger evidence and more practical implementation can help realize the full protective value of second-generation vaccines in pregnancy.
